# β-Oxidation in ghrelin-producing cells is important for ghrelin acyl-modification

**DOI:** 10.1038/s41598-018-27458-2

**Published:** 2018-06-15

**Authors:** Chika Ikenoya, Shota Takemi, Arisa Kaminoda, Sayaka Aizawa, Shiomi Ojima, Zhi Gong, Rakhi Chacrabati, Daisuke Kondo, Reiko Wada, Toru Tanaka, Sachiko Tsuda, Takafumi Sakai, Ichiro Sakata

**Affiliations:** 1 Area of Regulatory Biology, Division of Life Science, Graduate School of Science and Engineering, Saitama University, 255 Shimo-ohkubo, Sakuraku, Saitama, 338-8570 Japan; 2 Department of Biology, Graduate School of Natural Science and Technology, Okayama University, 3-1-1, Tsushimanaka, Kita-ku, Okayama, 700-8530 Japan; 3 Faculty of Pharmaceutical Sciences, Department of Pharmaceutical and Health Sciences, Josai University, 1-1 Keiyaki dai, Sakado, Saitama, 350-0295 Japan; 4 Research and Development Bureau, Saitama University, 255 Shimo-ohkubo, Sakuraku, Saitama, 338-8570 Japan; 5 Area of Life-NanoBio, Division of Strategy Research, Graduate School of Science and Engineering, Saitama University, 255 Shimo-okubo, Sakura-ku, Saitama, 338-8570 Japan

## Abstract

Ghrelin is a unique fatty acid-modified peptide hormone produced in the stomach and has important roles in energy homeostasis and gastrointestinal motility. However, the medium-chain fatty acid source for ghrelin acyl-modification is not known. We found that a fat-free diet and the removal of intestinal microbiota did not decrease acyl-ghrelin production in the stomach or plasma acyl-ghrelin levels in mice. RT-PCR analysis showed that genes involving fatty acid synthesis, metabolism, and transport were expressed in pancreas-derived ghrelinoma (PG-1) cells. Treatment with an irreversible inhibitor of carnitine palmitoyltransferase-1 (CPT-1) strongly decreased acylated ghrelin levels but did not affect ghrelin or ghrelin *o*-acyl transferase (GOAT) mRNA levels in PG-1 cells. Our results suggest that the medium-chain fatty acid used for the acyl-modification of ghrelin is produced in ghrelin-producing cells themselves by β-oxidation of long-chain fatty acids provided from the circulation.

## Introduction

Ghrelin is a 28-amino acid peptide hormone, which was identified by Kojima and colleagues in 1999^1^. This hormone is mainly released from the stomach and has many physiological activities such as stimulating food intake^2^, promoting growth hormone release^3^, modulating gastrointestinal motility^4,5^, decreasing blood pressure, and regulating energy metabolism^6^. In humans and rodents, plasma ghrelin levels increase in the fasting state and decrease immediately after eating^7^. It has also been reported that plasma ghrelin levels increased before each meal in humans who had a meal at determined time every day^8,9^, indicating that ghrelin plays an important role as a hunger hormone.

The structure of ghrelin is unique; the third serine residue of ghrelin is modified by medium-chain fatty acids (MCFAs) such as octanoic acid (C8) and decanoic acid (C10)^10^. Ghrelin is present in the blood and tissues in two forms: fatty acid-modified ghrelin called acylated- or acyl-ghrelin, and the unmodified form of ghrelin called des-acyl ghrelin. It has been well documented that this ghrelin modification is essential for the binding of ghrelin to its receptor (growth hormone secretagogue receptor; GHS-R) and the exertion of its physiological effects^11^. On the other hand, it has been reported that des-acyl ghrelin evokes some physiological functions, such as regulating body temperature^12^, increasing cardiac perfusion^13^, and impairing the orexigenic effects of ghrelin^14^, even though its receptor has not been identified^15^. In 2008, ghrelin *o*-acyl transferase (GOAT), an enzyme that specifically attaches MCFAs to ghrelin, was identified^16,17^. *Mboat4* knockout mice and ghrelin knockout mice showed similar phenotypes regarding body weight and blood glucose metabolism^18,19^. Moreover, a GOAT-specific inhibitor has been developed and the administration of this GOAT inhibitor decreased body weight gain^20^, indicating that targeting GOAT is a potential strategy to treat ghrelin-related issues such as energy imbalance or gastrointestinal disorders.

It has been found that feeding mice with chow containing heptanoic acid (C7) leads to the production of C7-modified ghrelin, which is not naturally synthesized *in vivo* in mammals^21^. Moreover, we previously found that the oral administration of octanoic acid to hatched chicks increased the amount of acyl-ghrelin in the stomach^22^. These results suggested that dietary MCFAs were directly used for ghrelin modification. However, the contribution of dietary MCFAs on ghrelin acylation is still unknown and other possible sources of MCFAs remain unclear.

Fatty acids are synthesized in the liver, adipose tissue, kidney, brain, lung, and mammary glands^23^. Specifically, MCFAs are synthesized in mammary glands, but it is expected that the amount of MCFAs from these organs is low^24^. On the other hand, fatty acid metabolic pathways that shorten acyl chains of long chain fatty acids (LCFAs) are also present and LCFAs and very long-chain fatty acids (VLCFAs) can be transferred from the cytoplasm into mitochondria or peroxisomes^25^. Recently, Bando *et al*. demonstarated that LCFAs are essential for acyl modification of ghrelin in a ghrelinoma cell line (MGN3-1)^26^. These LCFAs become medium-chain acyl-CoA by β-oxidation, and then medium-chain acyl-CoA is transferred from mitochondria or peroxisomes to the cytoplasm after being subjected to the action of acyl-CoA thioesterase (ACOT) or carnitine octanoyltransferase (CROT)^27–29^.

The aim of this study was to determine the source of MCFAs that modify ghrelin; thus, we examined the effect of diet, intestinal bacteria removal, and *de novo* synthesis or utilization of LCFAs in ghrelin-producing cells on acyl-ghrelin production in mice.

## Results

### Effect of a fat-free diet on acyl-ghrelin production in mice

**Figure 1:**
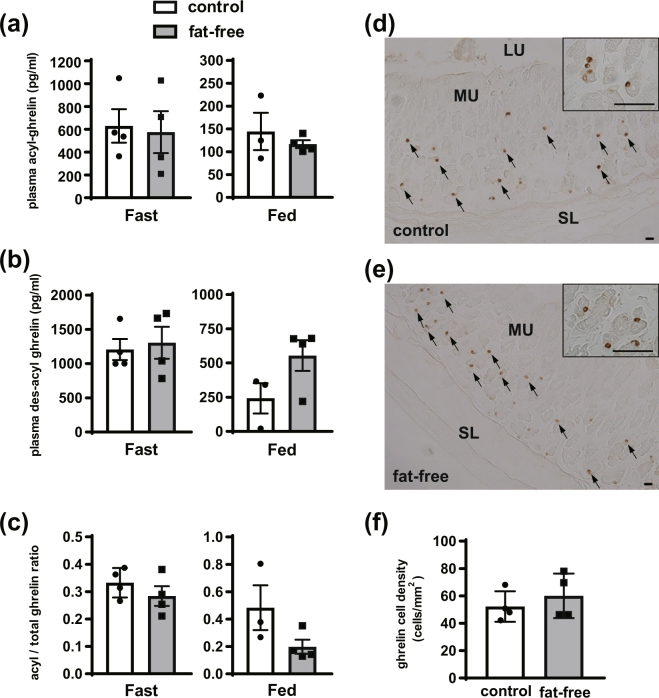
Effect of fat-free diet on ghrelin production in mice. After 3 weeks of feeding with the control or fat-free diet, plasma was collected at the fasting state (0900) and feeding state (1300). Plasma acyl-ghrelin levels **(a)**, des-acyl ghrelin levels **(b)**, and acyl/total ghrelin ratio **(c)** were not significantly different between control and fat-free groups. Staining intensities of acyl ghrelin-immunopositive cells from the control and fat-free group were similar (**d** and **e**). Ghrelin cell density in the gastric mucosa was not significantly different between the two groups **(f)**. This experiment was replicated twice and the results of 1st data set were used in this figure. MU, mucosal layer; SL, smooth muscle layer; LU, lumen. Scale bars = 25 µm (**d** and **e**). Each value represents the mean ± SEM. n = 4.

We first examined the effect of dietary fatty acids on ghrelin acylation. A fat-free diet was given to mice for 3 weeks after weaning (Fig. S1a). Although 3 weeks of fat-free diet intake tended to decrease body weight in comparison with control group mice (Fig. S2), plasma acyl-ghrelin concentrations were not altered between fasted and fed states (Fig. 1a). Des-acyl ghrelin concentrations and acyl/total ghrelin ratios also were not significantly different between groups (Fig. 1b,c). Immunohistochemical analysis using an antibody that recognizes acyl-ghrelin showed that the staining intensity of ghrelin-immunopositive cells in the gastric fundus was similar between control and fat-free groups (Fig. 1d,e). In addition, the number of ghrelin-immunopositive cells per unit mucosal area of the fat-free group was also comparable to the number in the control group (Fig. 1f).

### Effect of removing intestinal bacteria on acyl-ghrelin production in mice

**Figure 2:**
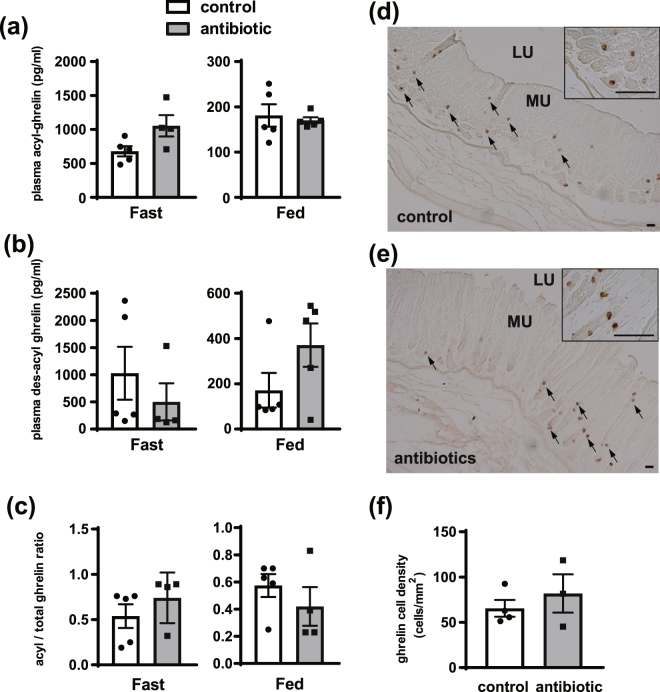
Effect of removing intestinal bacteria on ghrelin production in mice. After 5 days with or without administration of antibiotics, plasma was collected at the fasting state (0900) and feeding state (1300). Plasma acyl ghrelin level in the antibiotic group in the fasting state was higher than levels in the control group in the fasting state **(a)**. Plasma des-acyl ghrelin level and acyl/total ghrelin ratio were not significantly different between the control and antibiotic groups (**b**,**c**, respectively). Signal intensity and the number of ghrelin-immunopositive cell in the gastric mucosa did not change after antibiotic administration **(d–f)**. This experiment was replicated twice and the results of 2nd data set were used in this figure. MU, mucosal layer; SM, smooth muscle layer; LU, lumen. Scale bars = 25 µm **(d**,**e)**. Each value represents the mean ± SEM. n = 3–5. *P < 0.05.

Next, we tested the usage of the fatty acids produced from intestinal bacteria (Fig. S1b). To verify successful removal of intestinal bacteria in mice, feces were incubated on brain-heart infusion plates, and we confirmed that there were no colonies in antibiotic-treated mice (Fig. S3). Plasma acyl-ghrelin concentration was higher in the antibiotic-treated group than the concentration in the control group (Fig. 2a). However, plasma des-acyl ghrelin level and acyl/total ghrelin ratio was not significantly different between groups (Fig. 2b,c). The staining intensity of the ghrelin-immunopositive cells in the gastric fundus and the number of ghrelin-immunopositive cells per unit mucosal area were not significantly different between the antibiotic group and control group (Fig. 2d,e,f).

### Expression of genes involved in fatty acid synthesis and metabolism in PG-1 cells

**Figure 3:**
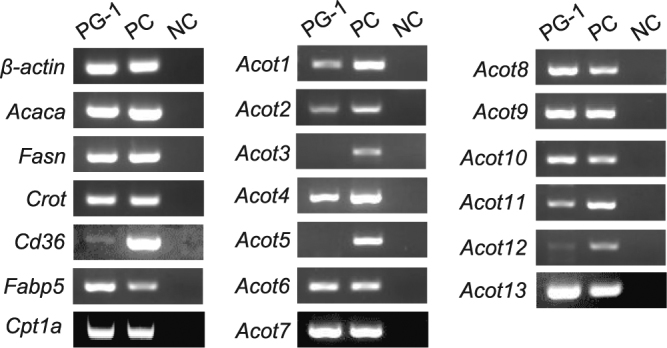
mRNA expression analysis by RT-PCR for genes involved in fatty acid synthesis and metabolism in PG-1 cells. Total RNA was extracted from PG-1 cells, cDNA was synthesized, then PCR was performed. PG-1 cells expressed acetyl-CoA carboxylase (*Acaca*) and fatty acid synthase (*Fasn*) from the fatty acid synthesis pathway. The mRNA expression of carnitine octanoyltransferase (*Crot*), CD36 antigen (*Cd36*), fatty acid binding protein 5 (*Fabp5*), and acyl-CoA thioesterase (*Acot*) *1*, *Acot2*, *Acot4*, *Acot6*, *Acot7*, *Acot8*, *Acot9*, *Acot10*, *Acot11*, *Acot12*, and *Acot13* were detected in PG-1 cells. PC, positive control: adipose tissue (*β-actin*, *Acaca*, *Fasn*, *Crot*, *Cd36*, and *Acot1*-*Acot11*) or liver (*Fabp5*, *Acot12*, and *Acot13*); NC, negative control (DW).

We then focused on *de novo* fatty acid synthesis in ghrelin-producing cells themselves, and examined the mRNA expression of fatty acid synthesis- and metabolism-related genes in PG-1 cells by RT-PCR. PG-1 cells expressed *Acaca*, *Fasn*, *Crot*, *Cd36*, *Fabp5*, *Cpt*1*a* and *Acots* mRNA but not *Acot3* and *Acot5* mRNA (Fig. 3). In addition, we identified protein production of CPT1A, FASN, and ACOT7 in PG-1 cell (Fig. S4).

### Effect of treating PG-1 cells with a FAS inhibitor (C75) and β-oxidation inhibitor (Etomoxir) on acyl-ghrelin production

**Figure 4:**
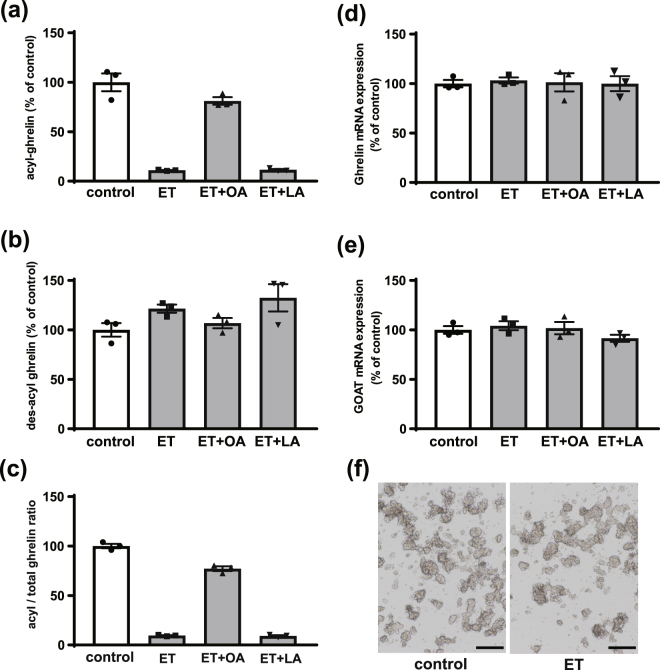
Effect of inhibiting β-oxidation on ghrelin production in PG-1 cells. PG-1 cells were plated in 24-well plates and etomoxir (ET, 5 µmol/L), octanoate (OA, 50 µmol/L), or a mixture of stearate and palmitate (LA, 50 µmol/L) was added. After incubation, acyl-ghrelin levels **(a)**, des-acyl ghrelin levels **(b)**, and acyl/total ghrelin ratio **(c)** in the medium were measured. Des-acyl ghrelin levels were not different between groups. ET treatment decreased acyl-ghrelin levels and the acyl/total ghrelin ratio, and ET + OA rescued acyl-ghrelin and acyl/total ghrelin ratio **(a–c)**. On the other hand, *Ghrl* and *Mboat4* mRNA expression was not changed between all experimental groups **(d**,**e)**. PG-1 cell morphology did not change after the etomoxir treatment **(f)**. This experiment was replicated twice and the results of 2nd data set were used in this figure. Scale bars=500 μm Each value represents the mean ± SEM. n = 3.

To determine the effect of fatty acid synthesis and β-oxidation on acyl-ghrelin production, we treated PG-1 cells with C75, a FAS inhibitor, and etomoxir, an irreversible carnitine palmitoyltransferase-1 (CPT-1) inhibitor. Although des-acyl ghrelin secretion was unchanged upon C75 treatment, acyl-ghrelin secretion showed a decreasing trend upon FAS inhibition compared to control (*P* = 0.07 by Kruskal-Wallis test) (Fig. S5a,b). C75 treatment decreased the medium acyl-ghrelin concentration, which is 307 pg/ml, whereas the control is 448 pg/ml. However, most acyl-ghrelin production was maintained (Fig. S5c). On the other hand, acyl-ghrelin production was dramatically decreased upon etomoxir treatment (Fig. 4a), and acyl-ghrelin production was rescued by co-treating with etomoxir and octanoate (Fig. 4a). LCFA treatment was not able to recover acyl-ghrelin production, however (Fig. 4a). Total ghrelin levels in culture medium were similar between all experimental groups (Fig. 4b), and the acyl/total ghrelin ratio showed the same tendency as acyl-ghrelin levels (Fig. 4c). On the other hand, etomoxir treatment did not change *Ghrl* and *Mboat4* mRNA levels in PG-1 cells (Fig. 4d,e) and PG-1 cell morphology was not altered after etomoxir treatment (Fig. 4f).

## Discussion

The third serine of the ghrelin peptide is modified by MCFAs and this acyl-modification is pivotal for the physiological functions of ghrelin. Since ghrelin plays important physiological roles in maintaining homeostasis, stable production of acyl-ghrelin is necessary. For this, a sustainable MCFA supply into ghrelin-producing cells is also required to produce sufficient amounts of acyl-ghrelin. On the other hand, des-acyl ghrelin inhibits ghrelin by impairing the orexigenic effects of ghrelin^14^, suppressing gastric emptying^30^, and disrupting gastric motility^31^. In addition, it has been reported that des-acyl ghrelin lowers mitochondrial reactive oxygen species production and altered metabolic condition^32^. Previous studies found that dietary MCFAs are directly utilized for ghrelin acylation^21,22^. Since the discovery of ghrelin, researchers thought that the source of MCFAs for ghrelin modification originated from the diet; however, the possible contribution of other sources remains unclear.

First, we examined the effect of a fat-free diet on acyl-ghrelin production in mice. After 3 weeks of feeding mice a fat-free diet, plasma acyl-ghrelin level and the number of ghrelin-immunopositive cells in the stomach of the fat-free group were comparable with levels in the control group. This result suggests that the involvement of diet on acyl-ghrelin production is relatively minor. Thus, we next examined the effect of fatty acids produced from intestinal bacteria in mice. It is general accepted that most fatty acids produced by intestinal bacteria are short-chain fatty acids (SCFAs)^33^. However, some bacteria produce MCFAs. Thus, we measured acyl-ghrelin production after the removal of intestinal bacteria and found that removing intestinal bacterial did not reduce acyl-ghrelin levels in the plasma or stomach. However, in Fig. 2a,the plasma acyl-ghrelin concentration in a fasted state increased with antibiotic treatment, suggesting that intestinal bacteria could modulate plasma acyl-ghrelin levels. Recently, it has been demonstrated that some intestinal bacteria stimulate peptide hormone secretion such as GLP-1^34^ and PYY^35^. Considering this function of intestinal bacteria, there is a possibility that intestinal bacteria could be associated with acyl-ghrelin secretion, but it is not thought to be the main source of acyl modifications of ghrelin because the acyl/total ghrelin ratio shown in Fig. 2c did not change with antibiotic treatment. We used mice of different ages in fat-free diet and antibiotic experiment, which may cause some differences in body condition, such as lipid metabolism, and the function and number of mitochondria. Although it is needed to take mice ages into account, acyl/total ghrelin ratio did not change between the control and treated groups in both fat-free diet and antibiotic experiment, indicating that feeding and intestinal bacteria are less relevant to acyl modification. Taken together, this finding suggests that MCFAs from *de novo* synthesis, which involves production from SCFAs or the breakdown of LCFAs, are utilized for ghrelin acyl modification.

In this study, we also used the pancreatic ghrelinoma cell line, PG-1. PG-1 cells express high levels of *Ghrl* and *Mboat4* mRNA, and the addition of octanoate into cell culture medium increases the production of acyl-ghrelin by these cells^36^. RT-PCR analysis showed that mRNAs for genes involved in fatty acid synthesis, transport, and metabolism were expressed in PG-1 cells, indicating that ghrelin-producing cells have the ability to produce MCFAs by elongation from citrate or breakdown from LCFAs in ghrelin-producing cells themselves. Thus, we examined the effect of *de novo* synthesis by fatty acid synthase and found that fatty acid synthase inhibition slightly decreased acyl-ghrelin production by PG-1 cells, suggesting the involvement of this enzyme in the ghrelin acylation process. It has been reported that acyl-ghrelin stimulates the expression of fatty acid synthase in several cell types, such as the rat hypothalamic neurons^37^ or human differentiated adipocytes^38^, indicating that there is a possibility of positive feedback regulation in PG-1 cells. However, acyl-ghrelin production remained mostly intact despite fatty acid synthase inhibition in this experiment.

LCFAs are shortened by β-oxidation in mitochondria and peroxisomes^25^, and fatty acids in the cytoplasm are transformed into acyl-CoAs and transported to mitochondria via carnitine palmitoyltransferase-1(CPT-1) or peroxisome directly, where they are finally subjected to β-oxidation^39,40^. In mitochondrial β-oxidation, LCFAs (C12-C20), MCFAs (C6–12) and SCFAs (<C6) are shortened, whereas LCFAs and VLCFAs (>C22) are shortened in peroxisomal β-oxidation^25^. To date, 13 types of acetyl-CoA transporters (ACOTs) have been identified, and ACOT2, 9, 10, and 13 are located in the mitochondria^41^. On the other hand, ACOT3, 4, 5, 6, and 8 are located in the peroxisome^41^. In this study, we found that *Acot2*, *Acot4*, *Acot6*, *Acot8*, *Acot9*, *Acot10*, and *Acot13* mRNA were expressed in PG-1 cells, suggesting that MCFAs are produced by β-oxidation from LCFAs and VLCFAs in ghrelin-producing cells. To test this, we treated PG-1 cells with a CPT-1 inhibitor, etomoxir. Etomoxir treatment dramatically reduced acyl-ghrelin production without affecting *Ghrl* and *Mboat4* mRNA levels. In addition, co-administration of etomoxir and octanoic acid rescued acyl-ghrelin production, however, co-administration of etomoxir and a LCFA did not rescue acyl-ghrelin production. These results strongly suggested that MCFAs from β-oxidation of LCFAs or VLCFAs are predominantly used for ghrelin acyl modification.

In conclusion, we demonstrated that MCFAs resulting from β-oxidation of LCFA are utilized for ghrelin acylation in ghrelin-producing cells *in vitro*. Although MCFAs from the diet can be used for ghrelin acylation, it is reasonable to consider that ghrelin-producing cells can utilize the supply of stored LCFA or LCFAs produced in the body without relying on food.

## Materials and Methods

### Animals

Male C57BL/6 J wild-type mice (Nihonikagaku, Tokyo, Japan) were used for these experiments. Animals were housed in plastic cages under controlled conditions (23 ± 2 °C, lights on from 0800 to 2000) with *ad libitum* access to food and drinking water. All procedures were approved and performed in accordance with the Saitama University Committee on Animal Research.

### Fat-free diet experiment

Male, 3-week-old mice were fed with the control diet (CE-2, CLEA Japan) or a fat-free diet (D04112303, Research Diets Inc., New Brunswick, NJ, USA) for 3 weeks. Mice were subjected to ad libitum feeding for the first 2 weeks, then feeding was restricted (fed from 0900 to 1300) for 1 week (Fig. S1a). After the 3 weeks, blood was collected from tail veins in the fasted state (0900) and feeding state (1300) to measure plasma ghrelin concentrations. Blood (100 µl) was collected in EDTA-coated tubes with p-hydroxymercuribenzoic acid (PHMB) at a final concentration of 1 mmol/L. Then blood was centrifuged at 4825 g for 10 min at 4 °C. The plasma was collected in a new tube and HCl was added to a final concentration of 0.1 N, and samples were stored at −80 °C until analysis. Mouse stomachs were fixed in 4% paraformaldehyde in PBS for immunohistochemistry. The experiment is repeated independently at least twice to confirm results.

### Removal of intestinal bacteria

Male, 10-week-old mice were fed with a fat-free diet and subjected to restriction feeding for 1 week (from Day 0 to 8). From day 2 to 6, mice were orally administered a mixture of antibiotics: ampicillin (017–10381, Wako, Osaka, Japan), kanamycin sulfate (6169400A1036, Meiji Seika Pharma, Kanagawa, Japan), vancomycin hydrochloride (226–01301, Wako), and metronidazole (132–18061, Wako) (10 mg of each antibiotic per mouse per day). On Day 7, drinking water was changed to water containing antibiotics (ampicillin, kanamycin sulfate, metronidazole: 1 g/L; vancomycin hydrochloride; 500 mg/L) to maintain sterile conditions (Fig. S1B). Blood and stomach tissue were collected as described above on Day 8. Feces of these mice were also collected to verify removal of intestinal bacteria. The experiment is repeated independently at least twice to confirm results.

### β-Oxidation inhibitor treatment in cells

PG-1 cells were plated at a concentration of 1 × 10^6^ cells/well in 12-well plates with DMEM/F12 50:50 (Cellgro, Manassas, VA, USA) supplemented with 10% fetal bovine serum, penicillin (100 U/mL), streptomycin (100 μg/mL), and etomoxir (E1905, Sigma-Aldrich, St. Louis, MO, USA) at a final concentration of 5 µmol/L. Simultaneously, sodium octanoate (C5038, Sigma-Aldrich) or mixture of sodium stearate and palmitate (199–03285, Wako) was added at a final concentration of 50 µmol/L and cells were incubated in a 37 °C incubator with 5% CO_2_ for 24 hours. After incubation, the medium was collected and centrifuged at 4135 g for 5 min at 4 °C. Next, 100 μL of supernatant was transferred to a separate tube and 10 μL of 1 N HCl was immediately added to the tube. Cells were collected in ISOGEN (Nippon Gene, Tokyo, Japan) for quantification of *Ghrl* and *Mboat4* mRNA. The collected samples were stored at −80 °C until analysis.

### Measurement of ghrelin concentration

Plasma and medium concentrations of acyl-ghrelin and des-acyl ghrelin were measured by an acylated Ghrelin (mouse, rat) Express EIA Kit (A05117, SPI Bio, Montigny-le-Bretonneux, France) and an Unacylated Ghrelin (mouse, rat) Express EIA Kit (A05118, SPI Bio). Acyl-ghrelin and des-acyl ghrelin concentrations were determined according to the manufacturer’s protocol. For acyl-ghrelin ELISA, the intra-assay coefficient of variation = 5.7%, and the inter-assay coefficient of variation = 6.1%. For des-acyl ghrelin ELISA, the intra-assay coefficient of variation = 5.2%, and the inter-assay coefficient of variation = 5.5%. Absorbance data were collected by using a Bio-Rad iMark Microplate Reader spectrophotometer, using Microplate Manager 6 Software (Bio-Rad, Hercules, CA, USA). Total ghrelin was calculated by adding acyl-ghrelin and des-acyl ghrelin concentrations together.

### Immunohistochemistry

After 24 hours of fixation, tissues were dehydrated with an ascending ethanol series, immersed in xylene, and then embedded in paraplast. Sections (10 µm thick) were made using a microtome and were mounted on silane-coated slides (Sigma-Aldrich). The sections were deparaffinized with xylene, rehydrated, treated with 0.5% sodium metaperiodate to block endogenous peroxidase for 10 min, treated with 1% sodium thiosulfate to clear residual iodine from sodium metaperiodate for 10 min, and then were incubated with blocking reagent (1% bovine serum albumin and 0.4% Triton X-100 in PBS) for 1 hour. After washing with PBS, sections were incubated with rabbit anti-ghrelin serum (#603)^42^ diluted 1:100,000 in blocking reagent for 18 hours. After washing with PBS, a second incubation with biotin-conjugated anti-rabbit IgG (Vectastain ABC kit; Vector, Burlingame, CA, USA) diluted 1:300 in blocking reagent was performed for 1 hour. Finally, the sections were incubated for 30 min with an ABC solution (Vectastain ABC kit). After washing in PBS for 15 min, sections were incubated in 0.02% 3,3-diaminobenzidine-tetrachloride (DAB) mixed with 0.006% H_2_O_2_ in 0.05 mol/L Tris-HCl (pH 7.6) for 3–5 min to detect immunostaining. After washing with water, the sections were dehydrated with a graded ethanol series, cleared in xylene, mounted with Entellan (Merck, Darmstadt, Germany), and viewed under a light microscope (BX60, Olympus, Tokyo, Japan). All procedures were performed at room temperature and incubations were carried out in a humidified chamber. After taking digital photographs of gastric fundi under a light microscope with a digital camera (DP70, Olympus), the number of acyl-ghrelin-positive cells in each section was counted and the area of the mucosal layer in each section was measured using Image J software (National Institutes of Health, Bethesda, MD). The ghrelin-producing cell density was calculated as the number of immunopositive cells per unit area. All data are expressed as mean ± SEM.

### RT-PCR to analyze the expression of fatty acid synthesis and metabolism genes in PG-1 cells

PG-1 cells were cultured in DMEM/F12 supplemented with 10% fetal bovine serum, penicillin, and streptomycin (Wako) in a 37 °C incubator with 5% CO_2_.

Total RNA was extracted from PG-1 cells and adipose tissue from 12-week-old male C57BL/6 J mice using ISOGEN (Nippon Gene). Trace DNA contamination was removed by DNase digestion, and cDNA was synthesized from 1 µg total RNA using the reverse transcriptase PrimeScript II (Takara Bio, Shiga, Japan). The primers were designed to amplify the mouse *β-actin*, *Acaca*, *Fasn*, *Acots*, *Crot*, *Cd36*, *Cpt1a* and *Fabp5* (Supplemental Table 1). PCR amplification was performed using Ex Taq polymerase (Takara Bio). Initial template denaturation was performed for 5 min at 94 °C. PCR cycles were programmed as follows: 30 sec at 94 °C (denaturation), 30 sec at 60 °C (annealing), and 30 sec at 72 °C (extension) for 40 cycles, followed by a final extension step for 10 min at 72 °C except for *Acaca*, *Fasn*, and *Cd36* (40 sec for each cycle step). Amplified cDNAs were run on 2% agarose gels and visualized by ethidium bromide staining using a UV illuminator (ATTO, Tokyo, JAPAN).

### Quantitative RT-PCR for *Ghrl* and *Mboat4* mRNA

After the extraction of total RNA, 0.5 µg of total RNA were used for cDNA synthesis using Primescript II Reverse Transcriptase (Takara Bio). Real-time PCR was performed using a LightCycler 96 (Roche Diagnostics, Indianapolis, IN, USA) with SYBR premix Ex Taq (Takara Bio) with the following primers: mouse *Ghrl* (fragment size: 67 bp): forward, 5′-CCCAGGCATTCCAGGTCAT-3′, reverse, 5′-AACTGCAGATGGTGCCTGAAG-3′; mouse *Mboat4* (fragment size: 71 bp): forward, 5′-TCCACAGCCTGGCTCTTTAAAC-3′, reverse, 5′-GCCGCGTGGAGGAGAGA-3′. The initial template denaturation was performed for 30 sec at 95 °C and PCR was performed with 45 cycles of 5 sec at 95 °C and 30 sec at 60 °C.

### Statistical analysis

All data are expressed as the mean ± SEM and statistical analyses were performed using GraphPad Prism 5 Software (La Jolla, CA, USA). Data were analyzed using a Mann–Whitney U test or kruskal-wallis test. Differences were considered statistically significant at *P* < 0.05.

## Electronic supplementary material

Supplementary Information File
