# Supplementary material for: β-Oxidation in ghrelin-producing cells is important for ghrelin acyl-modification
Source: Sci Rep. 2018 Jun 15;8:9176. doi: 10.1038/s41598-018-27458-2 (PMC6003948; doi:10.1038/s41598-018-27458-2)
Supplement: Supplementary file 1 — Supplementary Information File [file 41598_2018_27458_MOESM1_ESM.pdf]

Supplementary information for:

β-Oxidation in ghrelin-producing cells is important for ghrelin acyl-modification

[1]Chika Ikenoya[#], [1]Shota Takemi[#], [2]Arisa Kaminoda, [2]Sayaka Aizawa, [1]Siomi Ojima, [1]Zhi Gong, [1]Rakhi Chacrabati, [1]Daisuke Kondo, [1]Reiko Wada, [3]Toru Tanaka, [1, 4]Sachiko Tsuda, [1, 5]Takafumi Sakai, [1]Ichiro Sakata

[1]Area of Regulatory Biology, Division of Life Science, Graduate School of Science and Engineering, Saitama University, 255 Shimo-ohkubo, Sakuraku, Saitama 338-8570, Japan
[2]Department of Biology, Graduate School of Natural Science and Technology, Okayama University, 3-1-1, Tsushimanaka, Kita-ku, Okayama, 700-8530, Japan
[3]Faculty of Pharmaceutical Sciences, Department of Pharmaceutical and Health Sciences, Josai University, 1-1 Keiyaki dai, Sakado, Saitama, 350-0295, Japan
[4]Research and Development Bureau, Saitama University, 255 Shimo-ohkubo, Sakuraku, Saitama 338-8570, Japan
[5]Area of Life-NanoBio, Division of Strategy Research, Graduate School of Science and Engineering, Saitama University, 255 Shimo-okubo, Sakura-ku, Saitama 338-8570, Japan

Correspondence address:
Ichiro Sakata, Ph.D.
Area of Regulatory Biology, Division of Life Science, Graduate School of Science and Engineering, Saitama University, 255 Shimo-ohkubo, Sakuraku, Saitama 338-8570, Japan
Phone: +81-48-858-9117; Fax: +81-48-858-3422
E-mail: isakata@mail.saitama-u.ac.jp

#: These authors contributed equally

Evaluation of intestinal bacteria removal.

Feces were resuspended by PBS (1-2 pellets in 500 µl in sterilized PBS (-)). Then, feces were plated on brain heart infusion plate: mix Brain Heart Infusion Bacto (237400, BD) and Agar Bacto (214010, BD) followed by autoclave treatment (15 min at 121°C), Then defibrinated sheep blood was added amount to 10 % of the mixture. Plates were incubated for 48 hours at 37°C in anaerobic condition. After incubation, the number of colonies was evaluated.

Western blot analysis.

The protein expression of key genes identified by RT-PCR, including CPT1A, FASN, and ACOT7 were assessed by western blot analysis using PG-1 cell extracts. β-Actin protein was used as the housekeeping gene. PG-1 cells were collected and centrifuged at 1,200 ×*g* for 5 min at 4°C, the supernatant was removed, and the pellet was washed twice with cold PBS. RIPA buffer (0.2 mL) with 1X phosphatase inhibitor cocktail (Nacalai Tesque, Kyoto, Japan) was added, the cells were resuspended, and. the sample was placed on ice for 30 min. The cell lysate was centrifuged at 12,000 ×*g* for 10 min at 4°C, after which the supernatant was collected. Total protein concentrations were measured using a BCA™ Protein Assay kit (Thermo Fisher, Waltham, MA, USA), according to the manufacturer's instructions. 20 µg of protein (except for FASN; 10 µg) were run on 12% (except for CPT1A; 18%) SDS-PAGE and blotted onto PVDF membranes. The membranes were probed successively with anti-human β-Actin (no. PM053; Medical & Biological Laboratories [MBL] Co., Ltd, Nagoya, Japan) diluted 1:3,000, anti-human CPT1A (no. SC-393070; Santa Cruz Biotechnology, Inc., Heidelberg, Germany) diluted 1:3,000, anti-human Fatty Acid Synthase (no. sc-55580; Santa Cruz) diluted 1:100, and anti-human ACOT7 diluted 1:1,000 (no. sc-376808; Santa Cruz), followed by probing with horseradish peroxidase (HRP)-labeled antibody (Santa Cruz) diluted 1:10,000 (except for CPT1A; 1:20,000). The protein bands were visualized using Chemi-Lumi One L (Nacalai Tesque).

Supporting Figure Legends

Fig. S1. Experimental time schedules of fat-free diet (a) and removal of intestinal microbiota (b). After weaning, mice were fed a fat-free diet or control diet for 3 weeks. For 7 days before collecting plasma and tissues, mice were fed under a restricted feeding schedule (fed from 0900 to 1300) (a). Mice were fed with fat-free diet and subjected to restriction feeding for 1 week (from Day 0 to 8). From day 2 to 6, mice were orally administered a mixture of antibiotics (b).

Fig. S2. Effect of fat-free diet intake on mouse body weight. Three weeks of fat-free diet intake tended to decrease body weight compared to feeding with the control diet.

Fig. S3. Verification for the successful removal of intestinal bacteria. There were no colonies on the plate of antibiotic-treated mice, whereas colonies were observed on that of the control.

Fig. S4. Western blot analysis of ACOT7, FASN, and CPT1A in PG-1 cells.

Fig. S5. Effect of FAS inhibitor, C75, on ghrelin production in PG-1 cells. Plasma acyl-ghrelin levels tended to decrease upon C75 treatment ($P = 0.07$, Kruskal-Wallis test) (a). Des-acyl ghrelin levels were not influenced by C75 treatment (b). Acyl/total ghrelin ratio was

significantly decreased in C75-treated PG-1 cells, and C75 + OA treatment restored acyl/total ghrelin ratio (c). This experiment was replicated twice and the results of 2nd data set were used in this figure. Each value represents the mean ± SEM. n = 4. *P < 0.05. OA: octanoate.

Fig. S6. Reproducibility of Fig. 1 experiment.

Fig. S7. Reproducibility of Fig. 2 experiment.

Fig. S8. Reproducibility of Fig. 4 experiment.

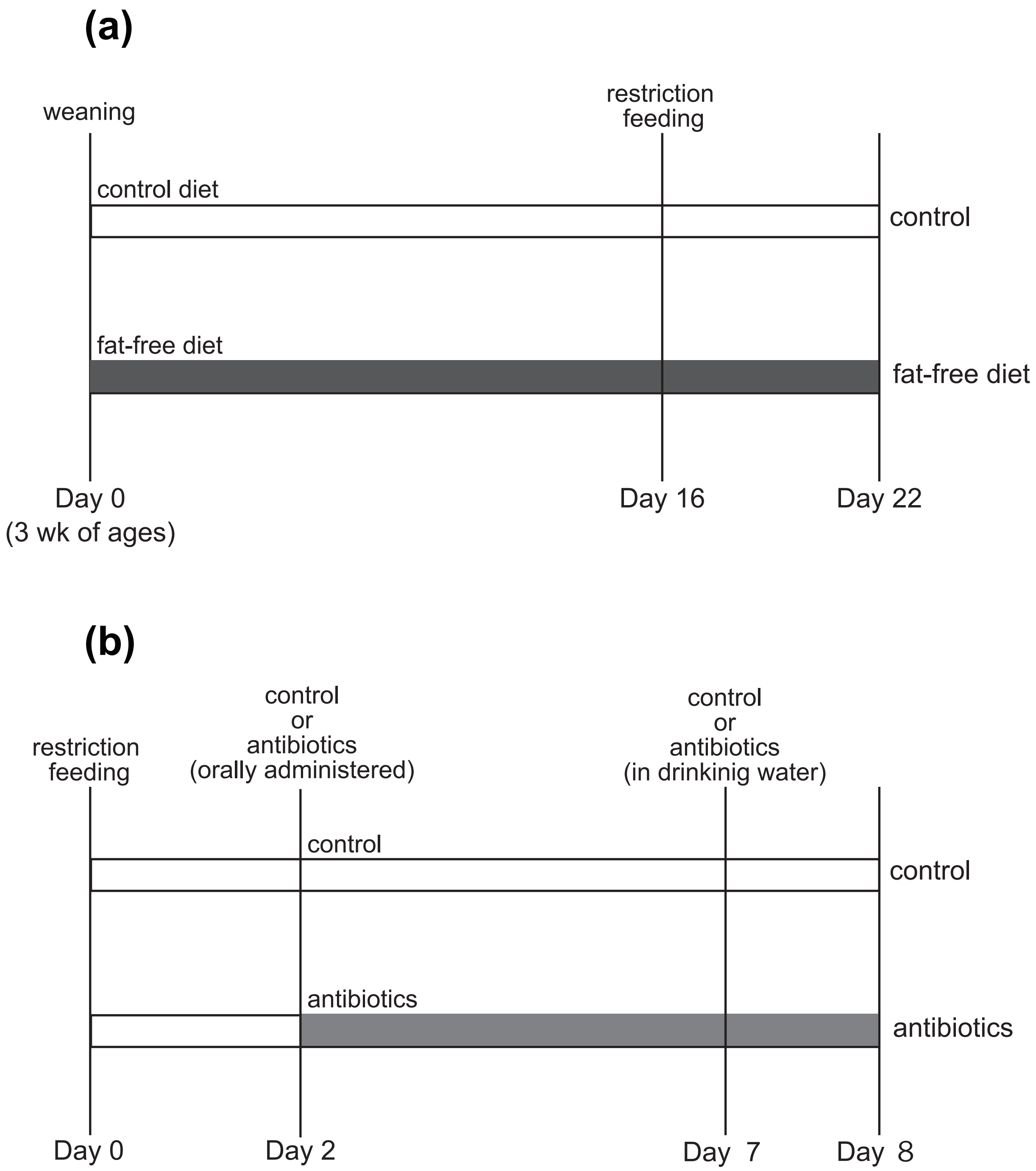
(a)
weaning
restriction feeding
control diet
control
fat-free diet
fat-free diet
Day 0
(3 wk of ages)
Day 16
Day 22
(b)
restriction feeding
control or antibiotics (orally administered)
control or antibiotics (in drinkinig water)
control
control
antibiotics
antibiotics
Day 0
Day 2
Day 7
Day 8

Fig. S1

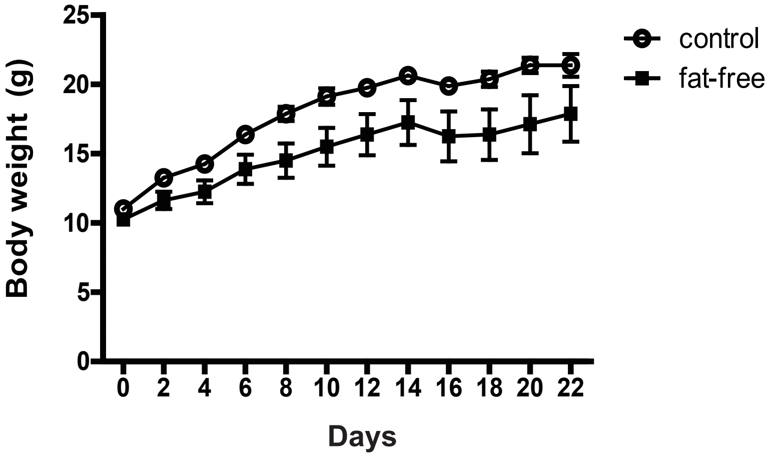

Body weight (g)
25
20
15
10
5
0
0 2 4 6 8 10 12 14 16 18 20 22
Days
control
fat-free

Fig. S2

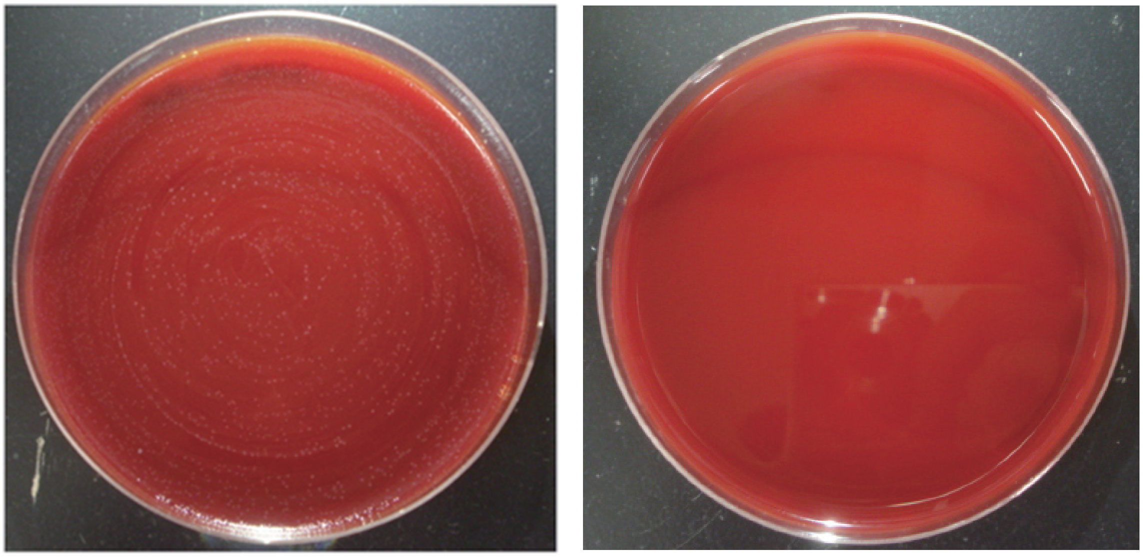

Fig. S3

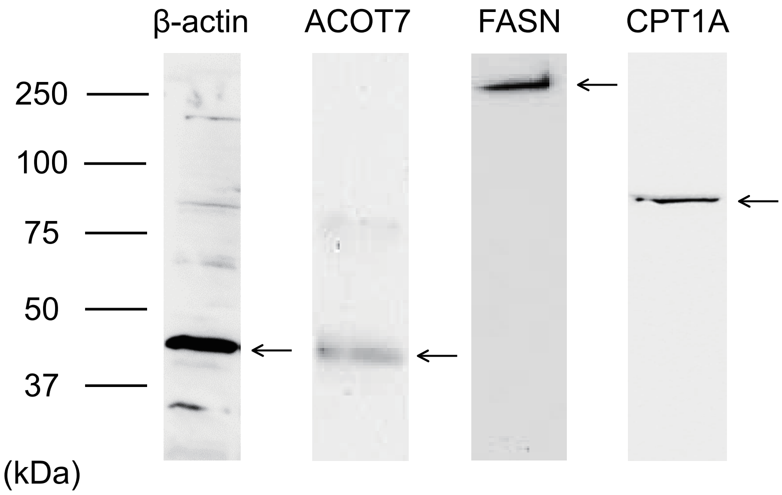

Fig. S4

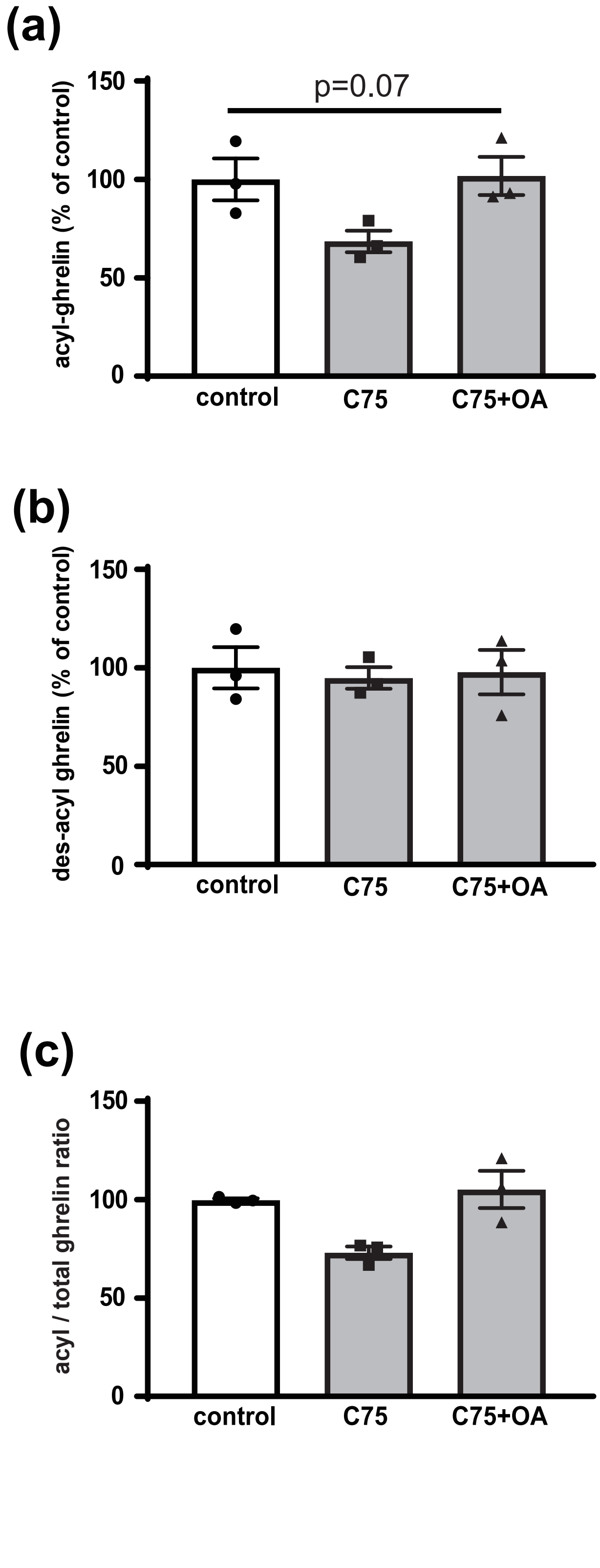
(a)
p=0.07
acyl-ghrelin (% of control)
150
100
50
0
control
C75
C75+OA
(b)
des-acyl ghrelin (% of control)
150
100
50
0
control
C75
C75+OA
(c)
acyl / total ghrelin ratio
150
100
50
0
control
C75
C75+OA

Fig. S5

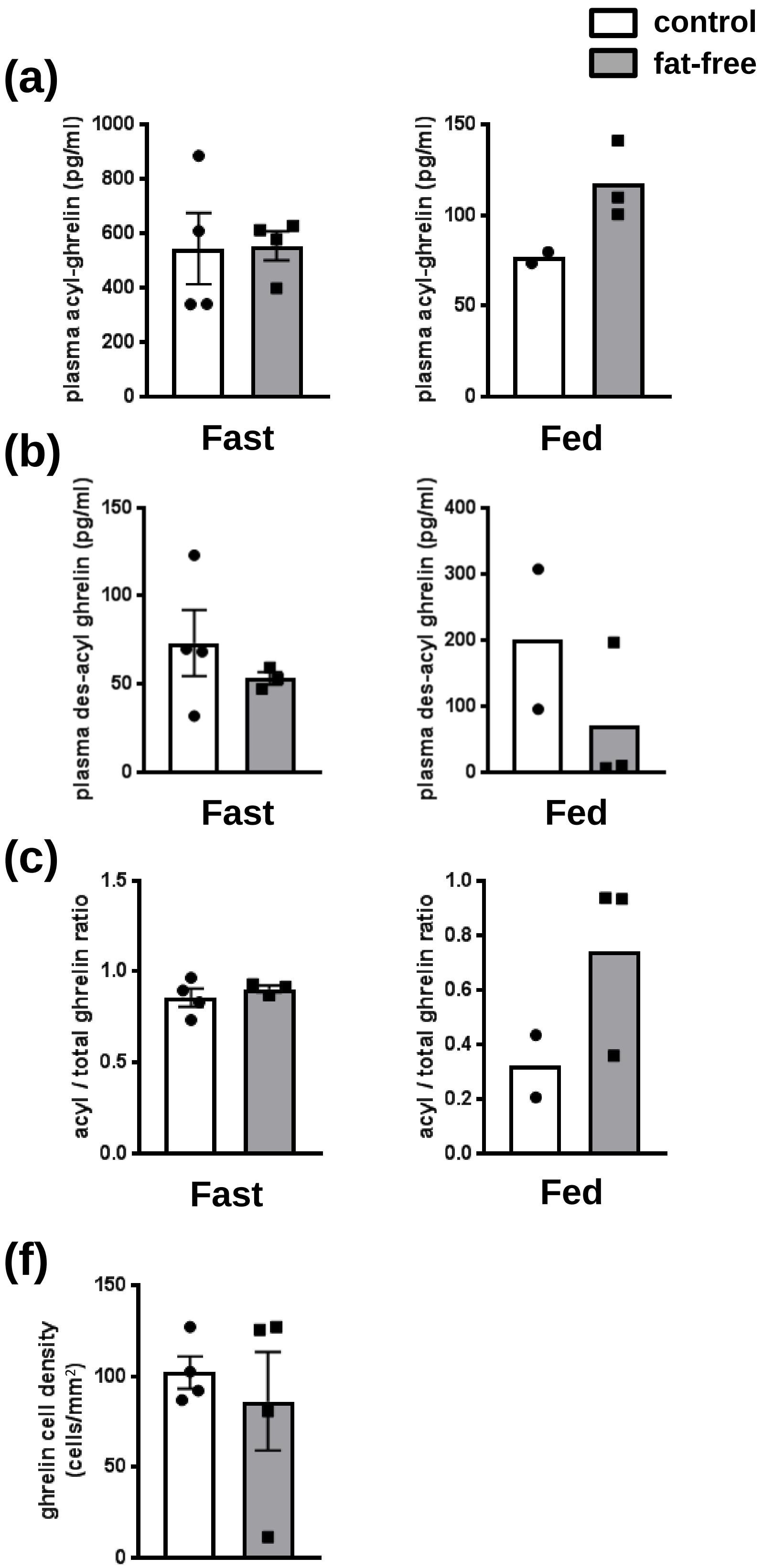
control
fat-free
(a)
plasma acyl-ghrelin (pg/ml)
1000
800
600
400
200
0
Fast
150
100
50
0
Fed
(b)
plasma des-acyl ghrelin (pg/ml)
150
100
50
0
Fast
400
300
200
100
0
Fed
(c)
acyl / total ghrelin ratio
1.5
1.0
0.5
0.0
Fast
1.0
0.8
0.6
0.4
0.2
0.0
Fed
(f)
ghrelin cell density (cells/mm2)
150
100
50
0

Fig. S6

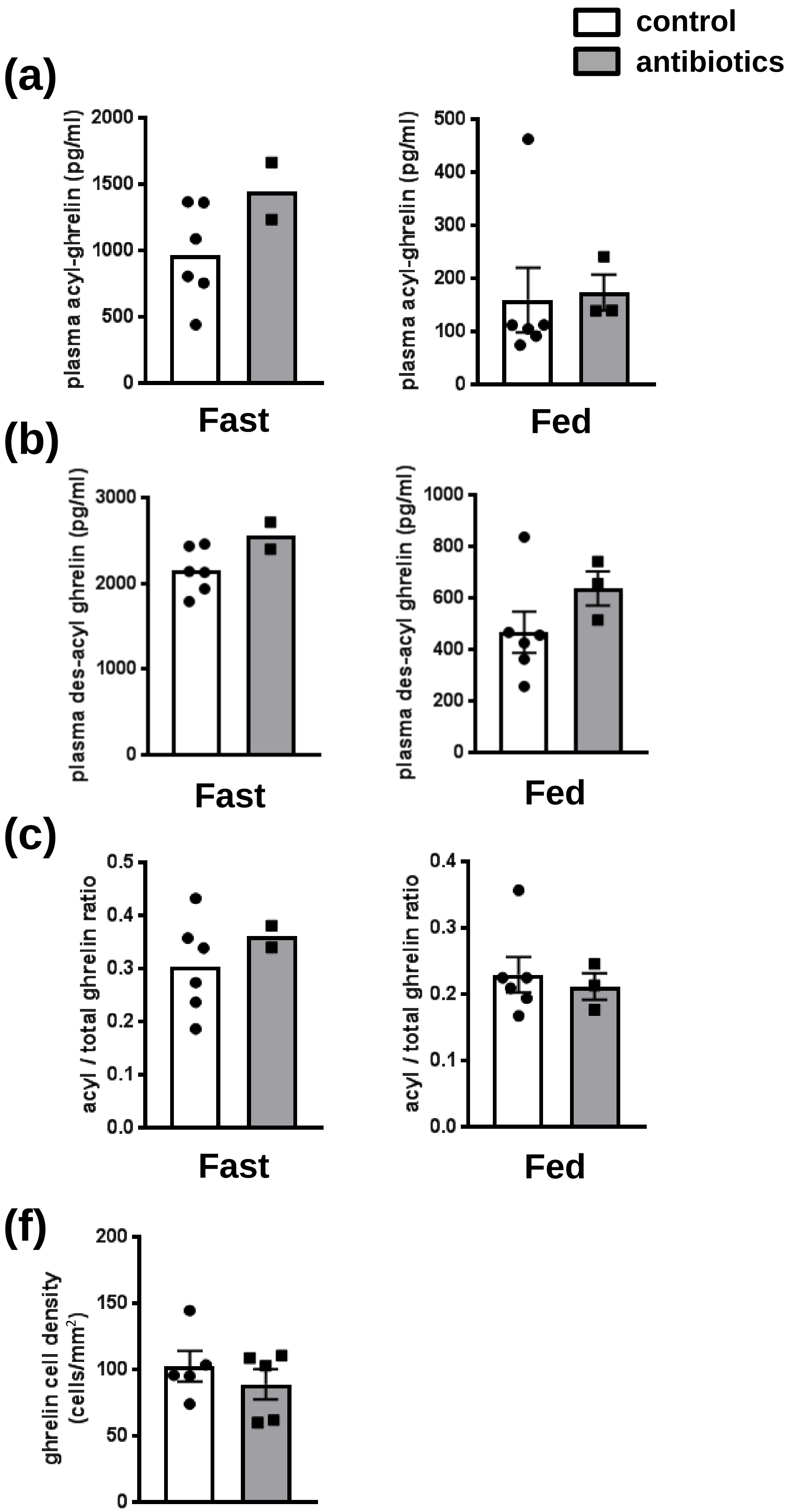
control
antibiotics
(a)
plasma acyl-ghrelin (pg/ml)
2000
1500
1000
500
0
Fast
500
400
300
200
100
0
Fed
(b)
plasma des-acyl ghrelin (pg/ml)
3000
2000
1000
0
Fast
1000
800
600
400
200
0
Fed
(c)
acyl / total ghrelin ratio
0.5
0.4
0.3
0.2
0.1
0.0
Fast
0.4
0.3
0.2
0.1
0.0
Fed
(f)
ghrelin cell density (cells/mm²)
200
150
100
50
0

Fig. S7

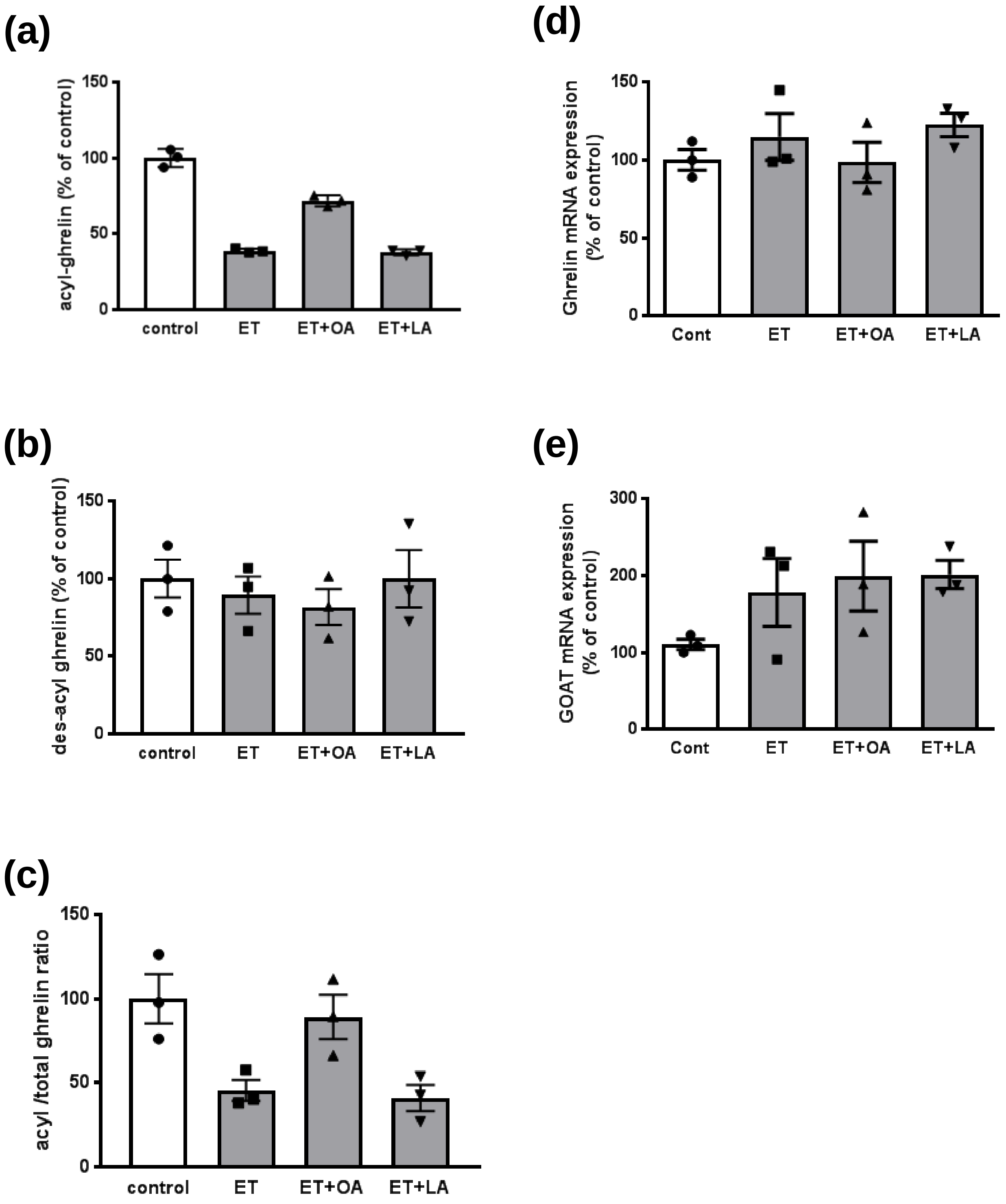
(a)
acyl-ghrelin (% of control)
150
100
50
0
control
ET
ET+OA
ET+LA
(b)
des-acyl ghrelin (% of control)
150
100
50
0
control
ET
ET+OA
ET+LA
(c)
acyl /total ghrelin ratio
150
100
50
0
control
ET
ET+OA
ET+LA
(d)
Ghrelin mRNA expression
(% of control)
150
100
50
0
Cont
ET
ET+OA
ET+LA
(e)
GOAT mRNA expression
(% of control)
300
200
100
0
Cont
ET
ET+OA
ET+LA

Fig. S8

Table S1. PCR primers for RT-PCR

Gene Name	Accession No.	Forward primer sequence	Reverese primer sequence	Size (bp)
β-actin	NM_007393.4	5’-CTGGGTATGGAATCCTGTGG-3’	5’-GTACTTGCGCTCAGGAGGAG-3’	213
Acaca	NM_133360.2	5’-AAATGACCCATCTGTAATGC-3’	5’-AGGCAGTATCCATTCATCAC-3’	363
Fasn	NM_007988.3	5’-TCAGCTATGAAGCAATTGTG-3’	5’-TATCCACTCCCTGAATCATC-3’	412
Crot	NM_023733.3	5’-GAGGAAGGTCTCCCTGTTCC-3’	5’-GATGAACATGCCACCACAAA-3’	194
Fabp5	NM_010634.3	5’-ATGGCAACAACATCACGGTC-3’	5’-CACACTCCACGATCATCTTCCC-3’	219
Cpt1a	NM_013495.2	5'-CTGAGCCATGAAGCCCTCAA-3'	5'-ACGCCACTCACGATGTTCTT-3'	257
Acot1	NM_012006.2	5’-CATGCACATGGAGTACTTTG-3’	5’-CTTGGTCATTTTGACCTGAT-3’	253
Acot2	NM_134188.3	5’-ATGGTTTGGGGGAAACCTCAT-3’	5’-TTGTCGTCTTACGGCACTGG-3’	376
Acot3	NM_134246.3	5’-ACTACGAGGACCTCCCTAAG-3’	5’-TCCTTCCAAAGGATTGTTTA-3’	332
Acot4	NM_134247.3	5’-CATCGATGTTTATGGTGTTG-3’	5’-GTTCTCACAGCCTCCTACAG-3’	436
Acot5	NM_145444.3	5’-TAGACCTTTTTGGAGTTGGA-3’	5’-TTGATCCTTTCTAGGTGCAT-3’	367
Acot6	NM_172580.1	5’-CTGGGATCATCGATTTGTAT-3’	5’-TTGGAGACACCAATAAGTCC-3’	226
Acot7	NM_001146057.1	5’-TAAGTCCATGGAGATTGAGG-3’	5’-GTGACTGCATACTGGGCTAT-3’	299
Acot8	NM_133240.2	5’-CGTAGAGAGGATACGGACAG-3’	5’-AATGCATCTTGATGTCACCT-3’	380
Acot9	BC021763.1	5’-ACCCTTACACATCCATCAAG-3’	5’-TGCATGTAGCAAACAAGAAC-3’	294
Acot10	NM_022816.2	5’-GGGCTCACGCTCACACTCTT-3’	5’-TATCTCCCGCAGCTTGACTTG-3’	225
Acot11	NM_025590.4	5’-AGAATGTGGGCAACCACTTG-3’	5’-CACCACTTGGCCGACACTAA-3’	326
Acot12	NM_028790.3	5’-CCGTGGCACTAAGGTCAGTT-3’	5’-GCTTGCCACATAAAAGGGCT-3’	332
Acot13	NM_025790.2	5’-GGCTTGCAGACTTGACCTTC-3’	5’-CATGGAGCGTGCCCAGTTTA-3’	265
